# Supplementary material for: Deep convolutional neural networks for segmenting 3D in vivo multiphoton images of vasculature in Alzheimer disease mouse models
Source: PLoS One. 2019 Mar 13;14(3):e0213539. doi: 10.1371/journal.pone.0213539 (PMC6415838; doi:10.1371/journal.pone.0213539)
Supplement: S1 Text — (PDF) [file pone.0213539.s001.pdf]

S1 Text. Manual 3D segmentation protocol using ImageJ

First, we created a new *hyper-stack* (File menu→New) with 3D voxel size and bit depth similar to the original image (e.g. a 16-bit $1024 \times 1024 \times 500$ voxel hyper-stack). The original image and the new hyper-stack were then merged (Image menu → Color) into a multi-channel hyper-stack, which contained both the raw data and the segmentation results.

On each image (in the x-y plane) the expert drew segmentation boundaries using the *free hand* tool and fill function (*F key*) while the second channel is selected using scrollbar. The *Color Picker* and *Channels Tool* (Image menu → Color) in addition to the Reverse CZT option (Edit menu → Options → Miscellaneous) were used to expedite the segmentation process.
