# Supplementary figures and images for: Deep convolutional neural networks for segmenting 3D in vivo multiphoton images of vasculature in Alzheimer disease mouse models
Source: PLoS One. 2019 Mar 13;14(3):e0213539. doi: 10.1371/journal.pone.0213539 (PMC6415838; doi:10.1371/journal.pone.0213539)

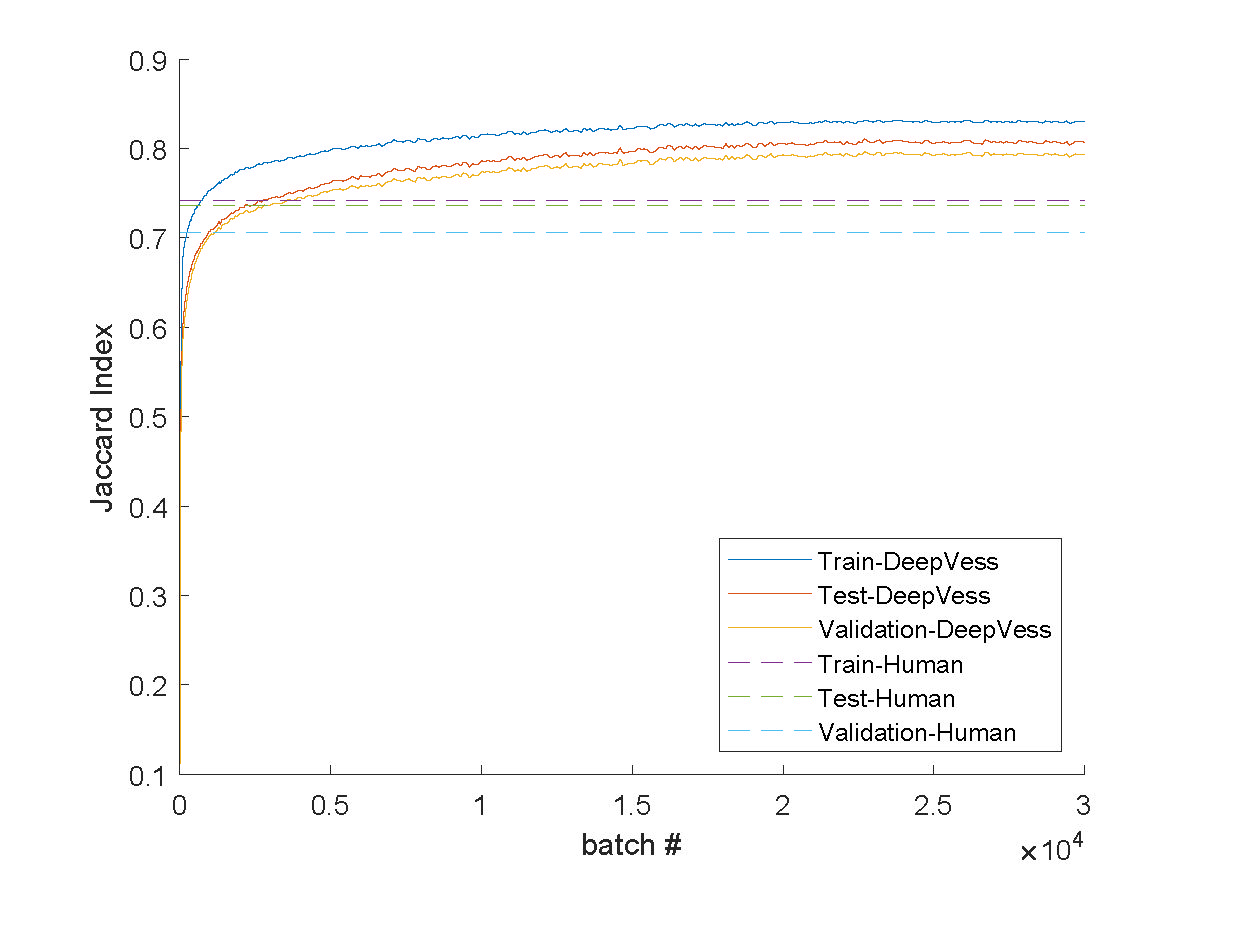

Supplement: S1 Fig — The DeepVess results surpass the trained human annotator result at all three train, validation, and test datasets. The human annotator and DeepVess results are shown in dashed and solid lines respectively. The constant difference between DeepVess and the human annotator’s results confirm the avoidance of overfitting. (TIF) [file pone.0213539.s002.tif]

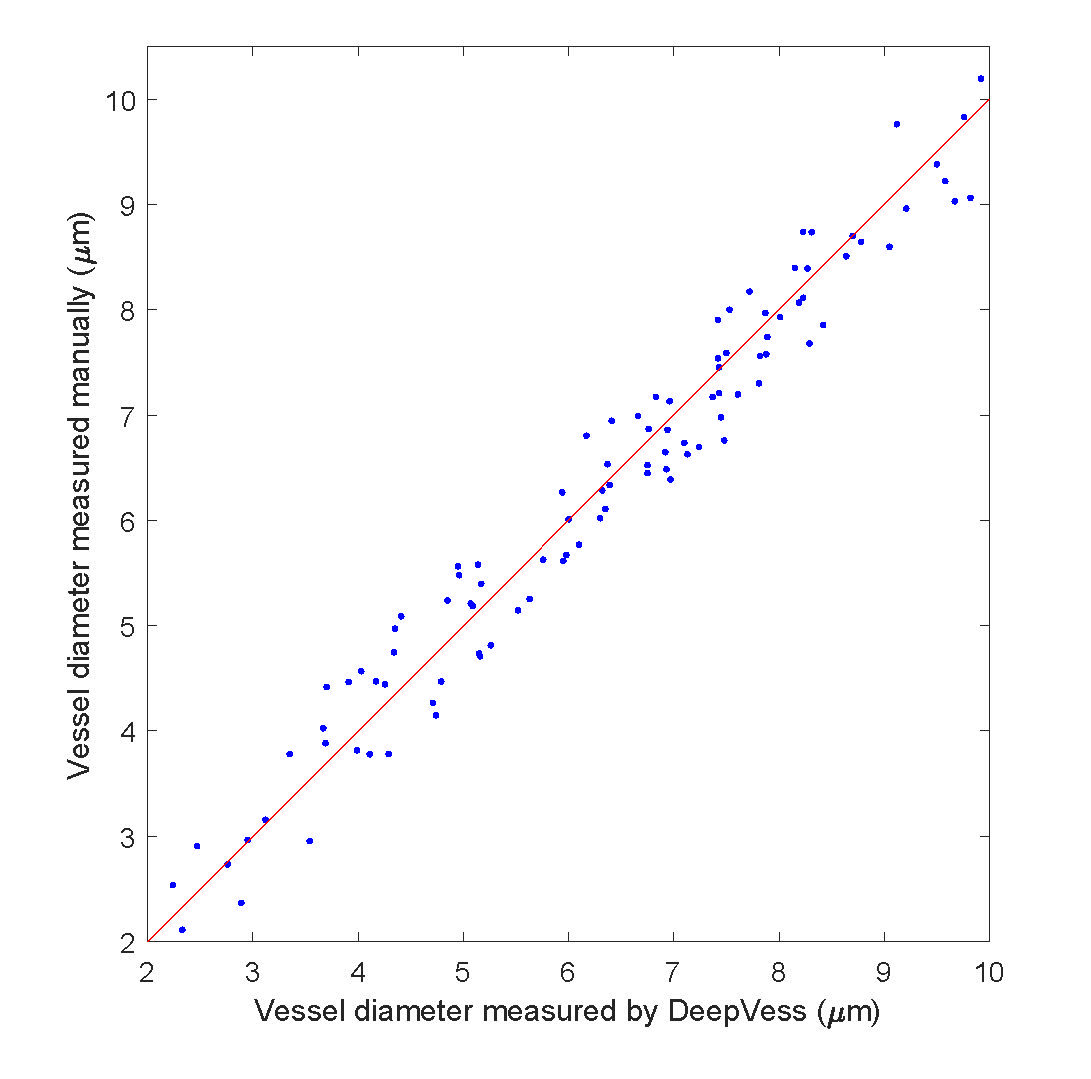

Supplement: S2 Fig — There is no significant difference between two measurements (paired t-test, n = 100, p = 0.34). (TIFF) [file pone.0213539.s003.tiff]
