# Supplementary material for: Deep convolutional neural networks for segmenting 3D in vivo multiphoton images of vasculature in Alzheimer disease mouse models
Source: PLoS One. 2019 Mar 13;14(3):e0213539. doi: 10.1371/journal.pone.0213539 (PMC6415838; doi:10.1371/journal.pone.0213539)
Supplement: S1 Table — (PDF) [file pone.0213539.s004.pdf]

S1 Table. The results of investigating different field of view sizes.

	Architecture	*FOV*
N1	C 7x7x5 - P - C 5x5 - P - NN	33x33x5
N2	C 7x7x9 - P - C 5x5 - P - NN	33x33x9
N3	C 7x7x15 - P - C 5x5 - P - NN	33x33x15
N4	C 7x7x31 - P - C 5x5 - P - NN	33x33x31
N5	C 7x7x5 - P - C 5x5 - P - NN	85x85x5
N6	C 7x7x7 - P - C 5x5 - P - NN	25x25x7
N7	C 7x7x7 - P - C 5x5 - P - NN	33x33x7
N8	C 7x7x7 - P - C 5x5 - P - NN	41x41x7
N9	C 9x9x9 - P - C 5x5 - P - NN	41x41x9

	Sensitivity	*Specificity*	*Dice*	*Jaccard*	*MHD*
N1	93.10%	98.15%	87.11%	77.17%	1.38
N2	87.39%	98.87%	87.40%	77.62%	1.15
N3	91.69%	98.31%	87.09%	77.13%	1.61
N4	89.94%	98.21%	85.69%	74.96%	2.19
N5	91.15%	98.23%	86.43%	76.11%	1.46
N6	90.22%	98.61%	87.71%	78.11%	1.03
N7	91.57%	98.49%	87.89%	78.40%	1.20
N8	91.01%	98.34%	86.86%	76.77%	1.85
N9	93.23%	97.61%	84.81%	73.63%	2.38
