# Supplementary material for: Deep convolutional neural networks for segmenting 3D in vivo multiphoton images of vasculature in Alzheimer disease mouse models
Source: PLoS One. 2019 Mar 13;14(3):e0213539. doi: 10.1371/journal.pone.0213539 (PMC6415838; doi:10.1371/journal.pone.0213539)
Supplement: S2 Table — (PDF) [file pone.0213539.s005.pdf]

S2 Table. The results of investigating different architectures.

	Architecture	***FOV***
N10	C 7x7x7 - P - C 5x5 - P - 2*NN	33x33x7
N11	3*C 3x3x3 - P - 3*C 3x3 - P - NN	33x33x7
N12	4*C 5x5x5 - P - 3*C 5x5 - P - NN	41x41x9
N13	4*C 3x3x3 - P - 3*C 3x3 - P - NN	41x41x9
N14	C 7x7x7 - P - C 5x5x5 - P - NN	25x25x25
N15	3*C 3x3x3 - P - 2*C 3x3x3 - P - NN	33x33x33
N16	3*C 3x3x3 - P - 2*C 3x3 - P - NN	41x41x41
N17	3*C 3x3x3 - P - 2*C 3x3 - P - NN	31x31x31
N18	3*C 3x3x3 - P - 2*C 3x3 - P - NN	49x49x49
N19	3*C 3x3x3 - P - 2*C 3x3 - P - NN	33x33x7
N20	previous architecture for ROI 5x5	33x33x7
N20P	previous architecture+post proc.	33x33x7

	Sensitivity	***Specificity***	***Dice***	***Jaccard***	***MHD***
N10	89.61%	98.33%	86.06%	75.53%	1.63
N11	93.71%	97.83%	86.00%	75.44%	1.87
N12	83.78%	98.68%	84.43%	73.05%	1.82
N13	93.45%	98.15%	87.30%	77.46%	1.48
N14	91.57%	98.49%	87.89%	78.40%	1.20
N15	90.29%	98.40%	86.77%	76.63%	5.98
N16	6.31%	93.76%	7.17%	3.72%	9.45
N17	14.82%	85.51%	10.71%	5.66%	9.48
N18	30.40%	72.32%	13.85%	7.44%	9.50
N19	92.89%	98.31%	87.74%	78.15%	1.16
N20	95.15%	98.40%	89.33%	80.71%	1.58
N20P	95.09%	98.47%	89.65%	81.24%	1.04
