# Supplementary material for: Deep convolutional neural networks for segmenting 3D in vivo multiphoton images of vasculature in Alzheimer disease mouse models
Source: PLoS One. 2019 Mar 13;14(3):e0213539. doi: 10.1371/journal.pone.0213539 (PMC6415838; doi:10.1371/journal.pone.0213539)
Supplement: S3 Table — (PDF) [file pone.0213539.s006.pdf]

S3 Table. The properties of six 3D images not used for training acquired from different mice included in the second independent dataset.

	Image Size (voxel)	***Voxel Size*** (μm^3)	***Z evaluation interval***	***Background***	***Trans gene***	***Phenotype***
1	$256 \times 256 \times 100$	$1.14 \times 1.14 \times 1$	1 μm	C57/BL6	APP/PS1	AD
2	$256 \times 256 \times 250$	$0.95 \times 0.95 \times 1$	25 μm	C57/BL6	APP/PS1	WT
3	$256 \times 256 \times 25$	$0.95 \times 0.95 \times 1$	1 μm	C57/BL6	APP/PS1	WT
4	$256 \times 256 \times 25$	$0.95 \times 0.95 \times 1$	1 μm	C57/BL6	APP/PS1	WT
5	$256 \times 256 \times 25$	$0.95 \times 0.95 \times 1$	1 μm	C57/BL6	APP/PS1	WT
6	$256 \times 256 \times 25$	$0.95 \times 0.95 \times 1$	1 μm	C57/BL6	APP/PS1	WT
