# Supplementary material for: Deep convolutional neural networks for segmenting 3D in vivo multiphoton images of vasculature in Alzheimer disease mouse models
Source: PLoS One. 2019 Mar 13;14(3):e0213539. doi: 10.1371/journal.pone.0213539 (PMC6415838; doi:10.1371/journal.pone.0213539)
Supplement: S4 Table — DeepVess surpass both of them in terms of sensitivity, Dice index, Jaccard index, and boundary modified Hausdorff distance (MHD). (PDF) [file pone.0213539.s007.pdf]

S4 Table. The results of *DeepVess* and the state-of-the-art methods on the second independent dataset from subjects not used for the model training (S3 Table). *DeepVess* surpass both of them in terms of sensitivity, Dice index, Jaccard index, and boundary modified Hausdorff distance (MHD).

	Sensitivity	***Specificity***	***Dice***	***Jaccard***	***MHD***
Teikari et al.	67.7%	99.3%	74.9%	60.6%	1.73
3D U-Net	72.4%	99.3%	78.5%	64.9%	1.45
DeepVess	**85.5**%	98.7%	**83.5**%	**71.8**%	**1.41**
